# Intentional- but not Unintentional Medication Non-adherence was Related with Beliefs about Medicines Among a Multi-Ethnic Sample of People with HIV

**DOI:** 10.1007/s10461-022-03842-y

**Published:** 2022-09-03

**Authors:** Anjuly Castelan, Jeannine F Nellen, Marc van der Valk, Pythia T Nieuwkerk

**Affiliations:** 1 Department of Medical Psychology, Amsterdam Public Health Research Institute, Amsterdam University Medical Centers, location AMC, University of Amsterdam, J3-219-1), Meibergdreef 9, 1105 AZ Amsterdam, the Netherlands; 2 Department of Infectious Diseases, Amsterdam University Medical Centers, Amsterdam Institute for Infection and Immunity, Amsterdam, the Netherlands

**Keywords:** HIV/AIDS, Combination antiretroviral therapy, Unintentional medication non-adherence, Intentional medication non-adherence, Beliefs about medicines

## Abstract

Medication non-adherence can be intentional or unintentional. We investigated the prevalence of unintentional and intentional non-adherence to antiretroviral therapy (ART) and the relationship with beliefs about medicines, sociodemographic- and HIV-related variables among people with HIV (PWH) attending the HIV clinic of the Amsterdam University Medical Centers. Participants completed the Medication Adherence Rating Scale (MARS) and the Beliefs about Medicines (BMQ) questionnaire. About half of 80 participants reported unintentional non-adherence and 20% reported intentional non-adherence. Both unintentional and intentional non-adherence were associated with younger age. Additionally, intentional non-adherence was associated with being a migrant from Suriname /Netherlands Antilles, having more concerns about negative effects of ART and stronger beliefs that medicines in general are overused/ overprescribed. In conclusion, intentional but not unintentional non-adherence was associated with beliefs about medicines. Eliciting and discussing beliefs about medicines may be a promising avenue to address patients’ concerns and perceptions thereby potentially enhancing medication adherence.

**Supplementary Information:**

The online version contains supplementary material available at 10.1007/s10461-022-03842-y.

## Introduction

Adherence to antiretroviral therapy (ART) is a primary determinant of antiretroviral treatment success. Sufficiently high levels of adherence to ART are necessary to achieve and sustain viral suppression thereby preventing morbidity and mortality [1–3]. Yet, people with HIV (PWH) do not always succeed in achieving or maintaining adequately high levels of treatment adherence [4]. Adherence is potentially amenable to intervention. To be able to intervene on low levels of medication adherence, problems with adherence need to be detected.

Non-adherence can be unintentional or intentional [5, 6], whereby intentional non-adherence is characterized by the patient’s active decision not to adhere to the prescribed medication. This is mainly influenced by perceptual barriers such as a perceived lack of necessity of medication or concerns about taking medication. Unintentional non-adherence is considered to occur more subconsciously and is mainly influenced by practical barriers such as a lack of capacity, resources and skills (e.g. forgetting, poor comprehension). The distinction between unintentional and intentional medication non-adherence is highly relevant because the different causes of these two types of non-adherence require different interventions to enhance medication adherence. For example, helping someone to remember taking medicines will not likely improve adherence if a person intentionally skips medication due to concerns about negative effects of medicines or disclosure concerns. Unintentional and intentional medication non-adherence have been extensively investigated among patients with various medical conditions [5, 6].

Despite the wealth of studies that have investigated adherence to ART to date, only a few studies have distinguished between unintentional and intentional non-adherence among PWH [7–15]. Nevertheless, these few studies have shown that unintentional- and intentional non-adherence appear to be two separate entities with each of them being associated with a different set of predictors/associated factors, including in studies for HIV treatment. Intentional non-adherence was generally associated with motivations, perceptions and beliefs, whereas unintentional non-adherence was more often associated with demographic- and HIV-treatment related factors.

Beliefs about medicines have emerged as an important factor influencing medication adherence among people with various medical conditions [17]. Beliefs about medicines can be divided into four themes: beliefs about the necessity of the prescribed medication, concerns about possible adverse effects of prescribed medication, beliefs that medicines in general are overused/over-prescribed by doctors, and beliefs that medicines in general are potentially harmful [16]. Previous studies have shown that stronger beliefs in the necessity of ART and lower concerns about ART are related with improved adherence [18–22]. However, none of these previous studies has distinguished between intentional and unintentional non-adherence.

The present study was conducted at the HIV clinic of the Amsterdam University Medical Centers location AMC in Amsterdam, the Netherlands. A substantial number of the persons receiving HIV care comprises non-western migrants. Non-western migrants living with HIV are a vulnerable group due to a combination of stigma surrounding HIV and, consequently, social isolation, frequent unfamiliarity with the Dutch health care system and poverty [23–25]. Non-western migrants were previously found to have lower levels of adherence to ART and a poorer health-related quality of life, as well as higher levels of experienced HIV stigma and depressive symptoms than PWH from other regions. High levels of HIV stigma and depressive symptoms mediated the relationship between migrant status on the one hand and low adherence and suboptimal viral suppression on the other hand [24].

This study was conducted during the corona pandemic, which might have influenced adherence to ART. Restrictive measures to curb the spread of SARS-CoV-2 could have introduced practical barriers to adherence such as difficulty with obtaining HIV medicines and having access to health care services, but also reduced social support and lead to feelings of loneliness, anxiety, and depression, which are known barriers to adherence. We were interested to learn to what extent PWH perceived that their level of adherence to ART had changed due to the corona pandemic and to what extent they perceived it to be more difficult to obtain their HIV medicines and receive medical care.

The present study’s aim was to investigate the prevalence of unintentional and intentional non-adherence to ART. The second aim was to identify associations between sociodemographic- and HIV-related factors, perceived changes in adherence and healthcare accessibility due to the corona pandemic, and beliefs about medicines on the one hand, and unintentional and intentional medication non-adherence on the other hand.

## Methods

### Patients

This cross-sectional study was conducted at the HIV outpatient clinic of the Amsterdam University Medical Centers location AMC, Amsterdam, Netherlands, from April to July 2021.

Consecutive persons attending the HIV clinic were asked to complete a questionnaire after their regular appointment with their infectious disease physician or nurse.

We included participants aged 18 years or older who were prescribed ART at the time of enrollment in this study. Exclusion criteria were (1) not being able to understand Dutch or English, or (2) no translator being present (such as family members and/or partner) in case the person was not able to understand Dutch or English. Illiterate persons who understood either Dutch or English were not excluded as the questions could be read aloud by the study coordinator (AC) if necessary. Participants were not compensated for their time. The study was exempted from formal ethics approval since the Dutch Medical Research Involving Human Subjects Act (WMO) does not apply (dd. April 6, 2021, W21_159#21.174).

## Medication Adherence and Beliefs about Medicines

The 5-item Medication Adherence Report Scale (MARS) was used to assess medication adherence [26–28]. One item asks about unintentional non-adherence (“I forget to take it”). Four items ask about intentional non-adherence (i.e.: “I decide to miss out on a dose”, “I take less than instructed”, “I stop taking my medications for a while” and “I alter the dose of my medications”). The MARS was specifically designed to minimize socially desirable responses by phrasing the questions in a non-judgmental manner that normalizes non-adherence. The items are scored on a 5-point scale ranging from “very often” to “never”.

Beliefs about medicines were assessed using the Beliefs about Medicines Questionnaire (BMQ) [16]. The BMQ was developed to enhance the understanding of patients’ perspectives on medication use. The BMQ consists of 19 items that form four subscales, i.e., the necessity (5 items), concerns (6 items), harm (4 items), and overuse (4 items) scales. The necessity and concerns scales ask about beliefs that specifically pertain to the prescribed HIV medicines, i.e., beliefs about the personal need for ART to maintain/improve current and future health and concerns about potential negative effects of ART on current or future health. The harm and overuse scales pertain to beliefs about medicines in general and measure the extent to which a person believes that medicines in general are harmful and overused/over-prescribed by doctors. BMQ items are described as statements that are answered on a five-point scale, ranging from 1 = strongly agree to 5 = strongly disagree. Scores of all BMQ items were reversed so that higher scores are indicative of stronger necessity beliefs, more concerns and stronger beliefs in harm and overuse. Scores obtained for the individual items within each scale are summed to give a scale score.

## Perceived Changes in Adherence and Healthcare Accessibility due to the Corona Pandemic

We administered the following three self-designed questions to assess perceived changes in adherence and healthcare accessibility due to the corona pandemic: (1) to what extent has the corona pandemic improved or worsened the way you take your HIV medicines? (response options: improved, no change, worsened), (2) to what extent has obtaining your HIV medicines become more difficult or easier since the corona pandemic? (response options: easier, no change, more difficult) and (3) to what extent has it become more difficult or easier for you to receive medical care since the corona pandemic, for example at the HIV outpatient clinic, at the pharmacy or from your general practitioner? (response options: easier, no change, more difficult).

Participants completed the MARS, BMQ and the questions about perceived changes due to the corona pandemic via the app Moniq (everywhereIM, Amsterdam, the Netherlands) that was installed on an iPad.

## Demographic and Clinical Background Characteristics

A number of demographic and clinical background characteristics were retrieved from the electronic medical file: age, gender, number of separate HIV medications (e.g. one single combination tablet or two/three separate tablets), daily dosing frequency, number of co-medications, time since HIV diagnosis, time since ART initiation, most recent viral load, and most recent CD4-cell count. Participants were asked to report their country of origin.

### Statistical Analysis

We calculated Cronbach’s alpha for the MARS total score, for the 4 MARS items measuring intentional non-adherence and for the necessity, concerns, overuse and harm scales of the BMQ. Cronbach’s alpha measures how closely related a set of items are as a group and values > 0.7 are considered acceptable.

Higher scores on the MARS indicate higher levels of medication adherence. We categorized patients into three groups based on their responses to the MARS items: (1) fully adherent patients (i.e., those who had the maximum score of 25 on the MARS-items), (2) patients reporting unintentional non-adherence (i.e., a below-maximum score on the MARS item ‘I forget to take it’ coupled with a maximum score on the four intentional MARS-items) and (3) patients reporting intentional non-adherence with or without unintentional non-adherence (i.e., a below-maximum score on the four intentional MARS-items combined with either a maximum or below-maximum score on the unintentional MARS-item).

We dichotomized responses to the questions about perceived changes in adherence and healthcare accessibility due to the corona pandemic into those reporting worsening in the way of taking HIV medicines versus no change or improvement, those reporting more difficulty in obtaining HIV medicines versus no change or easier, and those reporting more difficulty to receive medical care versus no change or easier.

Participants who were unable to complete the questionnaire by themselves and needed the study coordinator to read the questions aloud were categorized as having a major language barrier. Participants who needed a little help or explanation with answering the questionnaire were categorized as having minor language barriers. Participants who were able to complete the questionnaire independently were categorized as having no language barrier.

We investigated associations between reporting unintentional medication non-adherence versus being fully adherent (dependent variable) and demographic- and HIV-related characteristics, beliefs about medicines and perceived changes in adherence and healthcare accessibility due to the corona pandemic (independent variables) using logistic regression analyses. We also investigated associations between reporting intentional non-adherence with or without unintentional medication non-adherence versus being fully adherence (dependent variable) and demographic- and HIV-related characteristics, beliefs about medicines and perceived changes in adherence and healthcare accessibility due to the corona pandemic (independent variables) using logistic regression analyses. Two-sided p-values < 0.05 were considered statistically significant. All statistical analyses were conducted using SPSS version 26.

## Results

A total of 80 persons participated in the study. Persons attending the HIV clinic were asked by their HIV care provider if they were willing to participate during a regular visit to the outpatient clinic. Consenting persons were subsequently referred to the study coordinator. Consequently, the number of persons who had been asked but were not willing to participate was unknown. Characteristics of included participants are shown in Table 1. With respect to country of origin, the category “other” (6 participants), consisted primarily of South American participants and one East-Asian participant. One participant did not want to specify his/her country of origin.

**Table 1:** Characteristics of study participants

	Total sample *n* = 80
**Characteristic**	
Age (years), mean (SD)	52 (12)
Male gender, % (*n)*	64% (51/80)
Region/ country of origin^1^	
Netherlands	44% (35/80)
Africa	24% (19/80)
Suriname and Netherlands Antilles	11% ( 9/80)
Europe	13% (10/80)
Other	8% (6/80 )
Plasma HIV RNA concentration^2^	
< 50 copies/mL	95% (74/78)
50 to 100 copies/mL	1.3% (1/78)
100 to 1000 copies/mL	2.5% (2/78)
> 1000 copies/mL	1.3% (1/78)
Most recent CD4-cell count (cells/mm^3^), median (IQR)	680 (533 to 880)
Number of prescribed HIV-medications	
1	56% (45/80)
> 1	44% (35/80)
HIV medication daily dosing frequency	
1	85% (68/80)
> 1	15% (12/80)
Number of co-medications	
0	36% (29/80)
1	18% (14/80)
> 1	46% (37/80)
Years since HIV diagnosis, median (IQR)	17 (11 to 23)
Years since ART, median (IQR)	15 (9 to 21)

SD = standard deviation, IQR = interquartile range, 1 = one person did not want to disclose his/her region/ country of origin, 2 = missing for 2 persons

Participants included in the study were generally reflective of the entire population that is served by the HIV clinic of the AMC in terms of the percentage with suppressed viral load and the percentage with the Netherlands as country of origin, although the present sample included a slightly higher percentage of females and was slightly younger. For comparison, out of all individuals served by the AMC HIV clinic, a total of 2029 individuals were aged 18 years or older and had received ART for at least 6 months at the time of the present study. Of these individuals, 73% were males with a median age of 62 years, 27% were females with a median age of 56 years, 46% had the Netherlands as country of origin and 97.6% had plasma HIV RNA < 100 copies/mL.

## Medication Adherence

Chronbach’s alpha for the MARS was 0.73. Chronbach’s alpha for the 4 MARS items measuring intentional non-adherence was 0.85. Chronbach’s alpha for the BMQ necessity, concerns, overuse and harm scales were 0.83, 0.81, 0.57 and 0.62, respectively.

The median MARS score was 24 (IQR 23–25) indicating that adherence to ART was overall high as the maximum score on the MARS is 25. A total of 26 participants (32.5%) reported being fully adherent, i.e., they had a MARS score of 25. A total of 38 participants (47.5%) reported unintentional non-adherence without the presence of intentional non-adherence. A total of 16 participants (20%) reported intentional non-adherence with or without the presence of unintentional non-adherence. Only a single participant reported intentional non-adherence without unintentional non-adherence.

27 participants (33.8%) reported never forgetting to take their HIV medicines. 73 participants (91.3%) reported never altering the dose of their medication. 75 participants (93.8%) reported never stopping to take their HIV medicines for a while. 72 participants (90%) reported never deciding to miss out a dose of medication. Lastly, 73 participants (91.3%) reported never taking less medication than instructed.

## Perceived Changes in Adherence and Healthcare Accessibility due to the Corona Pandemic

A total of 6 participants (8%) reported worsening in their way of taking HIV medicines due to the corona pandemic, 5 (7%) reported more difficulty in obtaining HIV medicines and 17 (22%) reported more difficulty in receiving health care due to the corona pandemic.

## Sociodemographic and HIV-related Characteristics

Native language was Dutch for 43 participants, English for 8 participants and 29 participants had a native language other than Dutch or English. Ten participants (12.5%) had a major language barrier, out of which eight participants were African, one participant was European and one participant belonged to the category “other”. Two participants (2.5%) had a minor language barrier and needed some explanation of the questions. Due to the small size of the group “minor language barrier”, this group was later added to the group “no language barrier”.

## Factors Associated with Unintentional and Intentional Non-adherence

Differences between participants reporting full adherence versus participants reporting unintentional or intentional non-adherence in sociodemographic- and HIV-related-characteristics, perceived changes in adherence and healthcare accessibility due to the corona pandemic and beliefs about medicines are shown in Table 2.

**Table 2:** Factors associated with unintentional and intentional non-adherence

	Unintentional non-adherence (versus full adherence)		Intentional non-adherence (versus full adherence)	
	Odds ratio (95% confidence interval)	p- value	Odds ratio (95% confidence interval)	p-value
Age, years	0.94 (0.89, 0.99)	0.02	0.92 (0.86 to 0.99)	0.02
Male gender	Reference		Reference	
Female gender	1.10 (0.39 to 3.13)	0.86	1.13 (0.31 to 4.14)	0.85
**Country of origin** ^**1**^				
Netherlands	Reference		Reference	
Africa	0.96 (0.27 to 3.45)	0.95	2.71 (0.53 to 13.86)	0.23
Suriname and Netherlands Antilles	0.36 (0.03 to 4.42)	0.43	9.75 (1.38 to 68.78)	0.02
Europe	0.90 (0.20 to 4.03)	0.89	0.81 (0.07 to 9.52)	0.87
Other	3.61 (0.38 to 34.69)	0.27	*	1.00
No/minor language barrier	Reference		Reference	
Major language barrier	0.19 (0.03 to 1.01)	0.051	0.48 (0.08 to 2.71)	0.40
Most recent CD4-cell count	0.44 (0.07 to 2.82)	0.38	0.20 (0.01 to 3.03)	0.25
**Number of prescribed HIV-medications**				
1	Reference		Reference	
> 1	2.20 (0.79 to 6.10)	0.13	0.37 (0.08 to 1.63)	0.19
**HIV medication daily dosing frequency**				
1	Reference		Reference	
> 1	0.58 (0.14, to 2.48)	0.46	0.91 (0.14 to 6.16)	0.93
**No co-medication**	Reference		Reference	
1 **co-medication**	0.93 (0.21 to 4.18)	0.93	1.00 (0.16 to 6.26)	1.00
> 1 **co-medication**	0.61 (0.20 to 1.87)	0.39	0.68 (0.17 to 2.69)	0.57
**Years since HIV diagnosis**	1.02 (0.97 to 1.08)	0.42	0.99 (0.92 to 1.06)	0.76
**Years since cART**	1.00 (0.94 to 1.06)	0.98	0.97 (0.90 to 1.05)	0.46
**Beliefs about medicines**				
Necessity	1.03 (0.91 to 1.18)	0.61	0.89 (0.76 to 1.06)	0.19
Concerns	1.05 (0.94 to 1.18)	0.40	1.21 (1.04 to 1.41)	0.01
Overuse	1.09 (0.89 to 1.35)	0.40	1.32 (1.00 to 1.74)	0.05
Harm	1.12 (0.91 to 1.36)	0.28	1.24 (0.99 to 1.55)	0.06
**Perception of changes due to corona pandemic**				
Worsening of adherence	*		1.60 (1.10 to 2.34)	0.002
More difficult to obtain HIV medicines	3.04 (0.26 to 35.5)	0.72	0.48 (0.06 to 3.86)	0.60
More difficult to receive medical care	1.07 (0.31 to 3.73)	1.00	1.33 (0.30 to 5.96)	0.72

*Odds ratio could not be calculated due to empty cells/categories

Participants who reported unintentional or intentional non-adherence were significantly younger than participants who reported full adherence (51 years versus 57 years, t = 2,46, p = 0.02 and 48 years versus 57 years, t = 2,49, p = 0.02, respectively). Older age was significantly associated with a decreased likelihood to report unintentional non-adherence (OR 0.94 95% CI 0.89 to 0.99) and a decreased likelihood to report intentional non-adherence (OR 0.92 95% CI 0.86 to 0.99).

## Factors Associated with Unintentional Non-adherence

Participants with a major language barrier tended to report less unintentional medication-adherence compared to participants without a language barrier or a minor language barrier (20% (2/10) versus 51.4% (36/70), OR 0.19 95% CI 0.03 to 1.01, p = 0.051).

## Factors Associated with Intentional Non-adherence

**Figure 1:**
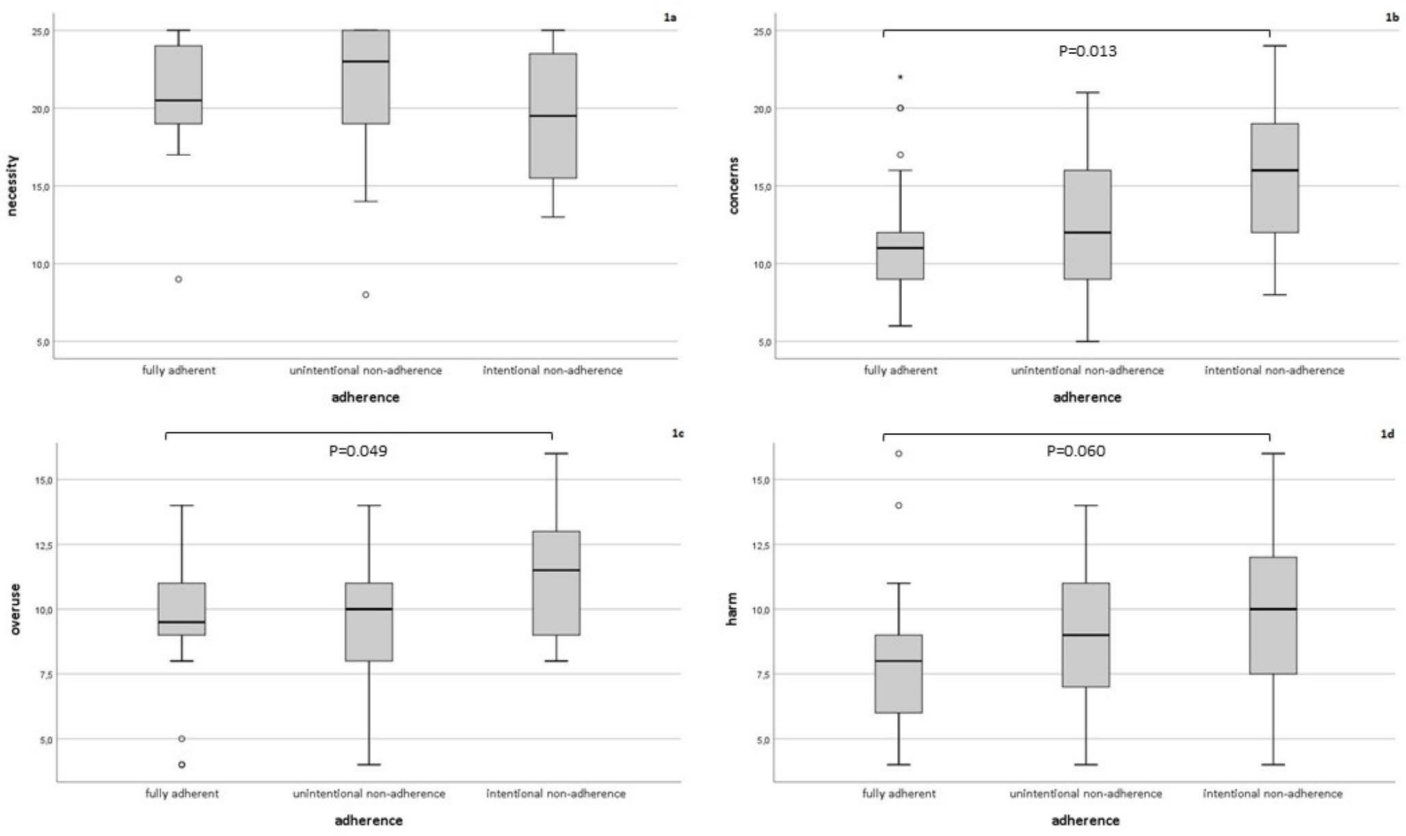
Fig. 1

Participants from Suriname or the Netherlands Antilles were significantly more likely to report intentional non-adherence than participants originating from the Netherlands (66.7% (6/9) versus 11.4% (4/35), OR 95% CI 1.38 to 68.78, p = 0.02). Participants reporting that the corona pandemic had worsened the way they took their HIV medicines were significantly more likely to report intentional non-adherence than participants reporting that their adherence had not changed or had improved the way they took their HIV medicines (37.5% (6/16) versus 0%, OR 1.60, 95% CI 1.10 to 2.34, p = 0.002). Participants who had higher concerns about potential negative effects of ART were more likely to report intentional non-adherent than participants having lower concerns about ART (OR 1.21, 95% CI 1.04 to 1.41, p = 0.01, Fig. 1B). Participants who had stronger beliefs that medicines in general are overused or overprescribed by doctors were more likely to report intentional non-adherence (OR 1.32, 95% CI 1.01 to 1.74, p = 0.05, Fig. 1 C) than participants with less strong beliefs. Participants who had stronger beliefs that medicines in general are harmful tended to be more likely to report intentional non-adherence (OR 1.24 95% CI 0.99 to 1.55, p = 0.06, Fig. 1D) than participants with less strong beliefs.

## Factors not Associated with Adherence

There were no statistically significant associations between adherence and gender, native language, most recent CD4-cell count, number of prescribed HIV medicines, daily dosing frequency, number of prescribed co-medications, years since HIV positive diagnosis, years since ART initiation, necessity beliefs (Fig. 1 A) and reporting more difficulty in obtaining HIV medicines or receiving health care due to the corona pandemic.

## Discussion

Among our multi-ethnic sample of PWH attending the HIV clinic of the Amsterdam University Medical Centers, about half reported unintentional non-adherence to ART, i.e. “forgetting”, and 20% reported intentional non-adherence to ART. Intentional but not unintentional non-adherence was associated with beliefs about medicines, specifically concerns about potential negative effects of ART and beliefs that medicines in general are overused/overprescribed by healthcare providers. Both unintentional and intentional non-adherence were associated with younger age.

Our findings are in line with previous research showing the influence of beliefs about medicines on treatment adherence across various chronic medical conditions [6, 16, 17, 35] as well in HIV treatment [18–21, 36]. The added value of the present study is that we distinguished between unintentional and intentional non-adherence to ART when examining the relationship with beliefs about medicines. We found that beliefs about medicines were related to intentional non-adherence but not to unintentional non-adherence. This distinction is relevant because different types of interventions will be required to overcome intentional non-adherence as compared with unintentional non-adherence. Intentional non-adherence would require intervening on perceptions and beliefs whereas unintentional non-adherence to ART would require addressing practical barriers such as forgetfulness, complexity of treatment and poor comprehension. Our findings suggest that eliciting and discussing beliefs about medicines may be a promising avenue to address patients’ concerns and perceptions of ART, thereby potentially enhancing medication adherence, which is important because PWH still face the challenge of having to take lifelong treatment.

Examples of strategies to explore and address people’s medicine-related beliefs in the clinical setting are the Perceptions and Practicalities Approach and the Necessity-Concerns Framework [17, 37]. These two strategies suggests that adherence support needs to address both the perceptions (e.g., beliefs about illness and treatment) and practicalities (e.g., capability and resources) affecting the individual’s ability and motivation to adhere. For many patients, taking medication does not ‘make common sense’ when they have an asymptomatic condition and feel well. These strategies include providing a rationale for medication necessity so that patients perceive a ‘common sense’ fit between their condition and treatment, eliciting and addressing concerns about medicines and addressing practical barriers to adherence. Exploring and addressing perceptual factors requires patient-centered communication. Carrying out non-judgmental assessments of what influences a person to want or not want to adhere and what factors influence their ability to do so is essential. Motivational interviewing is one technique which could be used to explore people’s perceptions of ART and foster their motivations to adhere.

Our finding that participants reporting non-adherence, either unintentional or intentional, were significantly younger than participants reporting full adherence is consistent with previous research [30–32] showing that older PWH have a lower risk of non-adherence than younger persons. Our findings thus corroborate that health care providers should be sensitive to adherence problems among younger persons. Since our participants comprised a relatively older population, i.e., mean age of 52 years, further research on unintentional and intentional non-adherence should be conducted among youth and young adults.

We found that migrants from Suriname or the Netherlands Antilles were more likely to report intentional non-adherence. Being part of an ethnic minority group was previously shown to be associated with lower medication adherence [33]. Risk factors for non-adherence, such as experiencing higher levels of HIV stigma, having higher levels of depression symptoms and experiencing lower levels of social support, are often more prevalent among migrants [23, 24]. Especially in African and Afro-Caribbean communities, stigma surrounding HIV is more prevalent [34]. However, African migrants did not report higher levels of non-adherence, either intentional or unintentional, compared with Dutch patients. Interesting to note is that Suriname and the Netherlands Antilles were former colonies of the Netherlands. Most of the Surinam or Antillean people living with HIV in the Netherlands are second generation migrants who have Dutch as their native language. In contrast, most African people living with HIV in the Netherlands are first generation migrants who often do not speak Dutch and are often less familiar with the Dutch health care system. Therefore, our finding that there was a difference in intentional non-adherence between Surinam/Antillean persons and Dutch persons, but not between African persons and Dutch persons in reported intentional non-adherence was unexpected. Possibly, this was due to small sample sizes of the different groups based on region of origin. Another explanation is that African patients may have been more inclined to provide socially desirable responses as eight of the African patients had the questions read aloud by the study coordinator due to illiteracy.

The level of reported intentional non-adherence in the present study was relatively low. This finding is consistent with previous studies showing that levels of reported intentional non-adherence are typically lower than levels of reported unintentional non-adherence [38]. Patients may be more inclined to report unintentional non-adherence than intentional non-adherence because forgetfulness is considered more socially acceptable than deliberately not taking medications [38].

A strength of the present study is that, because both Dutch and English versions of the questionnaire was available and because the questions could be read aloud by the study coordinator, participants who did not speak Dutch and participants who were illiterate could be included in the present study. This group of participants, mostly consisting of non-western migrants from resource-limited countries, are often excluded from studies because of language barriers and/or illiteracy. Another strength of the study is that we used two widely used and extensively validated instruments to assess medication adherence and beliefs about medicines.

This study also has limitations. Because of the cross-sectional design, no causal inferences can be made. This was a single center study from an HIV clinic serving a relatively large population of migrants living with HIV, mostly from sub–Saharan Africa. Therefore, our results cannot be generalized to the entire population of PWH in the Netherlands. Another limitation is the relatively small sample size. We used self-report as the adherence assessment method, which is known to overestimate medication adherence. The Chronbach’s alpha for the overuse and harm scales of the BMQ were below the commonly used threshold of 0.7, suggesting that not all items within these scales were closely related and/or that these scales included too few items. Results from these two scales should therefore interpreted with caution. Another limitation is that unintentional non-adherence was assessed with a single item. Although, in general, multi-item scales are considered to provide more reliable assessments than single item scales, there are several examples of single item scales that have successfully been used to measure adherence to ART [39–42]. Finally, we do not know how many potential participants were not asked by their health care provider to participate or refused to participate. For example, some health care providers mentioned not asking certain persons because they considered these persons to be poorly adherent and anticipated they would not be willing to participate in a study about adherence. Consequently, we cannot rule out the possibility of selection bias. However, the fact that the percentage of participants in the present study with suppressed viral loads was quite similar to that of the entire adult population who had received at least six months of ART at our HIV clinic argues against selection bias with respect to the level of adherence.

Our finding that beliefs about medicines are associated with intentional non-adherence is relevant for clinical practice as medication-related beliefs are in principle modifiable. We used patient-reported outcome measures (PROMS) to assess medication adherence and beliefs about medicines. PROMS are increasingly being used in individual clinical care to enhance communication between patients and healthcare providers about topics that are most relevant to patients and to identify patients in need of additional care [43]. Future studies should address whether the use of PROMs for assessing unintentional and intentional non-adherence would indeed improve discussions between patients and health care providers on the topic and facilitate the exploration of practical and perceptual barriers that patients experience, as well as considering patients’ beliefs in the provision of health care.

In conclusion, PWH reported both unintentional and intentional non-adherence to ART. Reporting intentional non-adherence to ART was significantly associated with beliefs about medicines. Eliciting and discussing beliefs about medicines may be a promising avenue to address patients’ concerns and perceptions thereby potentially enhancing medication adherence.

## Electronic Supplementary Material

Below is the link to the electronic supplementary material.

Supplementary Material 1
