# Supplementary material for: Intentional- but not Unintentional Medication Non-adherence was Related with Beliefs about Medicines Among a Multi-Ethnic Sample of People with HIV
Source: AIDS Behav. 2022 Sep 3;27(4):1045–54. doi: 10.1007/s10461-022-03842-y (PMC9440648; doi:10.1007/s10461-022-03842-y)
Supplement: Supplementary file 1 — Supplementary Material 1 [file 10461_2022_3842_MOESM1_ESM.docx]

**Appendix – Questionnaire**

***Part 1: BMQ-specific questions***

Instructions: We would like to ask you about your personal views about anti-HIV medication. These are statements other people have made about their anti-HIV medication. Please show how much you agree or disagree with them by ticking the appropriate box.

*Answer options: strongly agree, agree, uncertain, disagree, strongly disagree*

1. My health, at present, depends on these medicines

2. Having to take these medicines worries me

3. My life would be impossible without these medicines.

4. I sometimes worry about long-term effects of these medicines.

5. Without these medicines I would be very ill.

6. These medicines are a mystery to me.

7. My health in the future will depend on these medicines.

8. These medicines disrupt my life.

9. I sometimes worry about becoming too dependent on these medicines.

10. These medicines keep my HIV under control.

11. These medicines give me unpleasant side effects.

***Part 2: MARS***
Instructions: Many people find a way of using their HIV medicines which suits them. Please indicate for the following statements how much the apply to the way you take your HIV medication.

*Answer options: very often, often, sometimes, rarely, never*

12. I forget to take my medicines

13. I alter the dose of my medicines

14. I stop taking my medicines for a while

15. I decide to miss out a dose

16. I take less than instructed

***Part 3: BMQ-general questions***Instructions: We would like to ask you about your personal views about medicines in general. These are statements other people have made about medicines in general. Please indicate the extent to which you agree or disagree with them by ticking the appropriate box.

*Answer options: strongly agree, agree, uncertain, disagree, strongly disagree*

17. Doctors use too many medicines

18. People who take medicines should stop their treatment for a while every now and again

19. Most medicines are addictive

20. Natural remedies are safer than medicines

21. Medicines do more harm than good

22. All medicines are poisons

23. Doctors place too much trust on medicines

24. If doctors had more time with patients they would prescribe fewer medicines
